# DNA Double-Strand Break Repairs and Their Application in Plant DNA Integration

**DOI:** 10.3390/genes13020322

**Published:** 2022-02-09

**Authors:** Hexi Shen, Zhao Li

**Affiliations:** 1 School of Municipal and Environmental Engineering, Shandong Jianzhu University, Jinan 250101, China; lizhao19@sdjzu.edu.cn; 2 Resources and Environment Innovation Institute, Shandong Jianzhu University, Jinan 250101, China

**Keywords:** DSB, DNA repair, HR, NHEJ, T-DNA integration, gene targeting

## Abstract

Double-strand breaks (DSBs) are considered to be one of the most harmful and mutagenic forms of DNA damage. They are highly toxic if unrepaired, and can cause genome rearrangements and even cell death. Cells employ two major pathways to repair DSBs: homologous recombination (HR) and non-homologous end-joining (NHEJ). In plants, most applications of genome modification techniques depend on the development of DSB repair pathways, such as *Agrobacterium*-mediated transformation (AMT) and gene targeting (GT). In this paper, we review the achieved knowledge and recent advances on the DNA DSB response and its main repair pathways; discuss how these pathways affect *Agrobacterium*-mediated T-DNA integration and gene targeting in plants; and describe promising strategies for producing DSBs artificially, at definite sites in the genome.

## 1. Introduction

Double-strand breaks (DSBs) are considered to be one of the most harmful and mutagenic forms of DNA damage. They may arise as an outcome of normal cellular metabolism, but occur more frequently due to external factors such as ultraviolet (UV) radiation, ionizing radiation and genotoxic regents [1]. Being sessile organisms, plants are continually subjected to abiotic stress conditions, especially UV-B light and heavy metal pollutions, as well as to unexpected environmental changes that can also induce DNA damage [2]. DSBs are highly toxic if unrepaired, and can cause genome rearrangements and even cell death. Fortunately, cells possess highly conserved systems to recognize DSB signals and then trigger various downstream events to bring about repair. Two main DSB repair pathways are homologous recombination (HR) and non-homologous end-joining (NHEJ). The HR pathway recovers the genomic sequence precisely by using a template from the sister chromatid, the homologous chromosome or the homologous repeats in close proximity for accurate repair [3,4]. In contrast, the DSB ends are rejoined directly by the NHEJ pathway regardless of the sequence homology, leading to small deletions and insertions at the break site. HR and NHEJ are highly conserved in eukaryote cells, but their significance may be different depending on the cell type or the stage of the cell cycle. Unicellular eukaryotes mostly depend on HR to repair DSBs such as yeast *Saccharomyces cerevisiae* with small genomes, whereas the NHEJ pathway is the predominant one in higher eukaryotes such as humans and Arabidopsis, with large genomes containing many repeat sequences [5,6]. In plants, most applications of genome modification technique depend on the development of DSB repair pathways, such as *Agrobacterium*-mediated transformation (AMT) and gene targeting (GT). In this review, we summarize first the DNA DSB response and its main repair pathways. We also explore how these pathways affect *Agrobacterium*-mediated T-DNA integration and gene targeting in plants. In the last section, we discuss the strategies of producing site-specific DSBs artificially in the genome.

## 2. DNA Damage Response

The DNA damage response (DDR) is a signal transduction pathway that affects many aspects of cellular physiology (cell-cycle arrest, DNA repair, apoptosis and senescence) [7]. Key regulators of this pathway belong to the phosphatidylinositol 3-kinase-like protein kinase (PIKKs) family [8]. They control the downstream amplification of DNA damage signals by recruitment and phosphorylation of their substrates. The current understanding of the DDR mechanism in mammals is mostly dependent on the study of the three most important members of the PIKK family: ATM (Ataxia Telangiectasia Mutated), ATR (ATM and Rad-3 related) and DNA-PKcs (DNA-dependent protein kinase catalytic subunit) [9,10,11,12]. In human cells, MRE11-RAD50-NBS1 (MRN) complex and Ku70/80 heterodimer recognize DSBs signals and then trigger the activation of ATM and DNA-PKcs, respectively. Subsequently, the levels of DNA repair proteins are activated and/or induced. In addition, the appearance of areas in the genome with ssDNA as a consequence of DNA damage or repair leads to the recruitment and activation of the other master regulator, ATR. Afterwards ATM and ATR phosphorylate lots of downstream substrates, including CHK1 and CHK2, which generate a downstream amplification by protein activation and repression. These signals are subsequently transmitted to the tumor-suppressor protein p53, resulting in cell-cycle arrest and DNA repair. Another example includes the histone protein H2AX. Local phosphorylation of histone H2AX at damage sites leads to local accumulation of repair proteins, which is enhanced by ubiquitination and poly (ADP)-ribosylation of specific damage response pathway components. Multiple studies in human cells have concluded that ATM phosphorylates histone H2AX and chromatin remodeling factor KAP1 with DNA-PKcs, due to the functional redundancy between ATM and DNA-PKcs in the process [13,14,15]. However, a recent report discovered that DNA-PKcs responds to IR-mediated DSBs very quickly, and its enzymatic activity is able to initiate the DDR directly [16].

**Figure 1:**
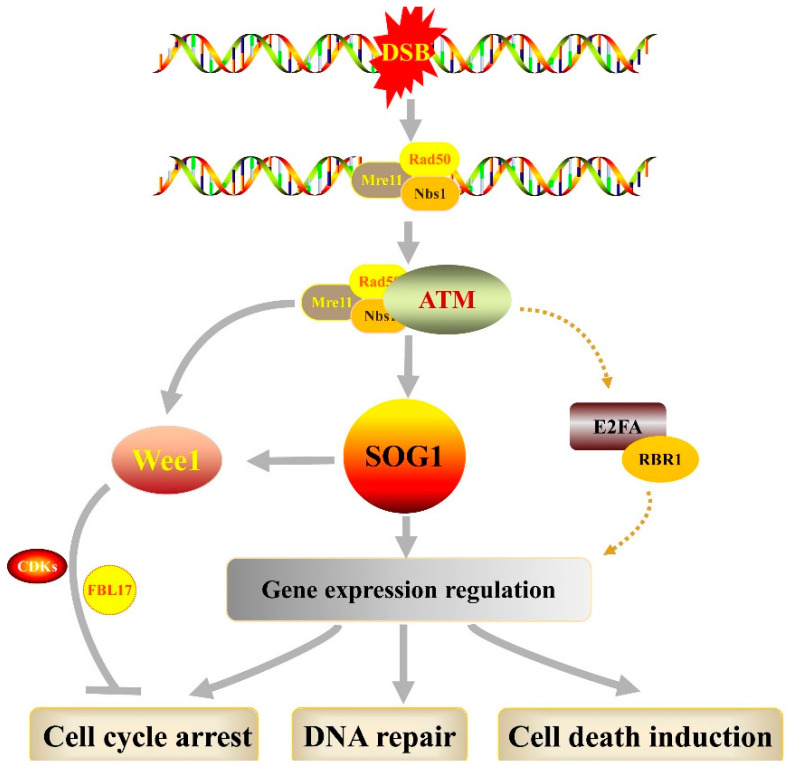
Overview of DNA Double-strand break (DSB) damage response in plants. MRN (MRE11-RAD50-NBS1) complex first recognizes the DNA DSB resulting in ATM (Ataxia Telangiectasia Mutated) activation. Subsequently, the ATM amplify signals by phosphorylation of downstream substrates, such as SOG1 (suppressor of γ response 1). The SOG1 functions as a central hub and controls the expression of hundreds of genes, which ultimately induce cell-cycle arrest, DNA repair or programmed cell death.

Plant DDRs are not fully understood in detail, even though the main actors of the DDR signaling pathway have been identified. So far, only a few homologous genes in plants have been characterized that are related to DDR. The Arabidopsis ATM and ATR proteins are the key DNA damage sensors for the response to DNA damage [17,18]. The MRN complex recognizes DSBs in plant cells and then activates ATM activity. Despite the conservation nature of ATM and ATR kinases in plants and animals, they seem to behave distinctively differently under DNA stress. Many genes required for the cell cycle and DNA damage checkpoints in animals have no ortholog in plants, such as CHK1, CHK2 and p53 [19]. Instead, a downstream factor called SOG1 (suppressor of γ response 1) functions as a central hub in the Arabidopsis DDR process [20,21]. As a plant-specific transcription factor, SOG1 controls the expression of most of the genes related to γ irradiation which ultimately induce cell-cycle arrest, DNA repair or programmed cell death [22,23]. In addition to being a target of SOG1, WEE1 is another critical downstream target of the ATR-ATM signaling cascades, and regulates cell-cycle arrest directly by phosphorylating and inhibiting CDKs, and/or indirectly by phosphorylating FBL17 in response to DNA damage [24,25]. Furthermore, recent findings revealed that the E2FA-RBR1 (retinoblastoma-related 1) complexes are responsible for the activation of the cell cycle checkpoint and likely function in the plant DDR in a SOG1-independent manner [26,27,28]. The role of E2FA-RBR1 complexes in DDR relies on ATM/ATR and CYCB1/CDKB activity, but the exact molecular mechanisms remain to be determined (Figure 1).

## 3. DSB Repair via Homologous Recombination

**Figure 2:**
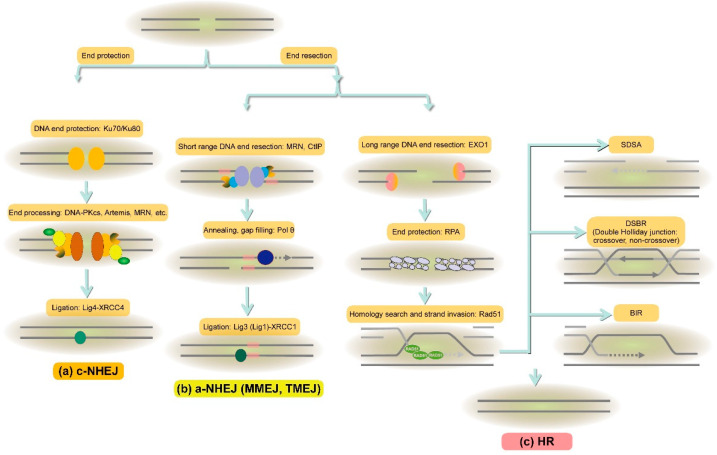
The major pathways of DNA DSB repair: (**a**) During the c-NHEJ, DSB is recognized and bound by Ku70/80. The binding of Ku70/80 can protect the DSB ends from end resection, followed by the recruitment of other factors to perform end processing. The Ligase4-XRCC4 complex executes the final ligation step; (**b**) in a-NHEJ (MMEJ or TMEJ) pathways, PARP1 is supposed to serve as a platform at the broken end to recruit other factors, including the DNA polymerase θ (Pol θ) which utilizes short microhomologies (indicated as pink boxes) for efficient and processive DNA synthesis. The microhomology-mediated joints between the two DNA ends are stabilized by Pol θ and work as primers for gap filling, while the XRCC1-Ligase3 (Ligase1) complex is responsible for the final ligation step; (**c**) HR is initiated by the long-range DNA end resection involving Exo1 and MRN complex. Subsequently, ssDNA overhangs are coated by RPA for protection against winding of the DNA. The Rad51 filament then facilitates a fast and efficient homology search and DNA strand invasion, resulting in a D-loop structure. One of three sub-pathways of HR complete the repair in the end: synthesis-dependent strand annealing (SDSA), double-strand break repair (DSBR) or break-induced replication (BIR) (does not exist in plants).

Homologous recombination (HR) promotes genome stability by facilitating the error-free repair of DSBs, interstrand crosslinks (ICLs), and DNA gaps, during and after DNA replication [29]. HR is a key repair pathway in the S and G2 phases of the cell cycle. HR pathway requires homologous sequences that act as a template, and the homologous sister chromatid is the preferred template in somatic cells. The information from the homologous sister chromatid is copied into, and replaces, the damaged region, resulting in precise repair. The homology search and DNA strand invasion are major steps in this process. Both are catalyzed by DNA-dependent ATPase Rad51, which can bind cooperatively to ssDNA to form helical nucleoprotein filaments [30]. HR consists of three stages, and Rad51 functions in all of them: pre-synapsis, synapsis and post-synapsis [31] (Figure 2).

At the beginning of pre-synapsis, the DSB is processed to generate ssDNA overhangs by 5′ to 3′ DNA end resection involving the Exo1 and MRN complex. In yeast, the MRX (MRE11-RAD50-XRS2) complex and Sae2 assist the short resection (50–200 nt), while more extensive resection involves either Exo1 or Sgs1 in combination with Dna2 [32]. After end resection, ssDNA overhangs are coated by RPA to protect against DNA degradation and the formation of secondary structures, which is required for the formation of competent Rad51 filaments [33]. However, RPA binding also prevents Rad51 filament assembly [34]. This inhibitory effect can be conquered by at least three different kinds of mediator proteins: Rad51 paralogs, Rad52, and BRCA2 [29]. In Arabidopsis, most of the major players in HR are identified and characterized [35,36] (Table 1).

The Rad51 filament facilitates a fast and efficient homology search and DNA strand invasion, resulting in a D-loop structure. Rad54 is required for searching for homology, stimulates DNA strand invasion by the Rad51 filament, and also functions after synapsis [37,38]. DNA synthesis is primed by the 3′ invading strand, using the donor strand as a template.

After extension of the 3′ invading strand, repair is finalized by one of at least three different sub-pathways of HR. In the classical double-strand break repair (DSBR) sub-pathway, Rad52 and Rad59 stimulate the arrest of the second end by the D-loop, whereafter two Holliday junctions (dHJ) are formed [39]. These dHJs are either dissolved into non-crossover products by a RecQ helicase, such as yeast Sgs1, or human BLM helicase, or decomposed into crossover/non-crossover products by a structure-specific endonuclease. In general, non-crossovers are much more predominant in mitotic HR [40]. Crossovers are able to produce genomic rearrangements and large-scale loss of heterozygosity (LOH) [41]. The Mph1 helicase suppresses the DSBR pathway to avoid crossover events in mitotic cells by dissociating D-loop formations, to promote annealing of the extended 3′ end to complementary sequences at the other side of the DSB, resulting in the synthesis-dependent strand annealing (SDSA) sub-pathway [42]. It seems that the Rad51 protein has some inhibitory activity that counters the capture of the second end and dHJ formation, indicating an inherent mechanistic bias toward SDSA [43]. The D-loop can convert into a replication fork in the absence of a second end, leading to the break-induced replication (BIR) sub-pathway. During BIR, the replication fork restores the integrity of a broken chromosome by copying the whole distal arm of the template chromosome, producing LOH [44]. However, no experimental evidence has been shown of the occurrence of BIR in plants [45].

## 4. DSB Repair via Non-Homologous End-Joining

Non-homologous end-joining (NHEJ) plays a major role in the repair of plant and mammal DSBs [5,6]. The NHEJ process seems to be relatively simple and straightforward: it rejoins broken ends directly, without the requirement of long runs of end-resection and searching for a homologous repair template. NHEJ pathways are subdivided into the canonical NHEJ (c-NHEJ) and a group of less well elucidated alternative NHEJ (a-NHEJ) pathways, which are also called microhomology-mediated end-joining (MMEJ) or polymerase theta-mediated end-joining (TMEJ) [46,47,48]. Still, many factors are required for NHEJ pathways that demand precise cooperation and timely regulation (Table 2). In general, DSB repair by NHEJ can be precise, but may also cause small nucleotide deletions and insertions at the junction, which changes the nucleotide sequence information surrounding the repair region [49]. As a result, NHEJ is considered as an error-prone DNA repair pathway. Our previous studies in Arabidopsis NHEJ-deficient mutants indicated that a-NHEJ is a more error-prone mechanism compared to c-NHEJ [50].

During the c-NHEJ pathway, DSB is recognized and bound by a Ku70/80 heterodimer to initiate the NHEJ repair [51]. The crystal structure of the human Ku heterodimer shows that the Ku heterodimer forms a ring structure on DNA broken ends in a sequence-independent manner [52]. Ku is an abundant protein that has an extraordinary affinity for dsDNA ends allowing it to quickly localize to DSBs [51,53]. The binding of Ku70/80 could protect the DSB ends from end resection, followed by the recruitment of other factors to perform end processing. Therefore, more NHEJ factors are subsequently recruited to perform end processing, including DNA-PKcs, Artemis, polynucleotide kinase/phosphatase (PNKP), the gap-filling DNA polymerases mu (Pol µ) and lambda (Pol λ), and the Mre11/Rad50/Nbs1 (MRN) complex. Arabidopsis Pol λ promotes DNA end processing in association with the XRCC4 and Ligase 4 [54]. The Ligase 4-XRCC4 complex executes final ligation of the broken ends [55].

In the absence of the Ku complex, human and plant cells still can accomplish end-joining by alternative pathways (Figure 2). The a-NHEJ pathways require elements of HR end-resection machinery, and is often associated with short tracts of microhomology different from c-NHEJ. The molecular mechanism of a-NHEJ initiation remains unclear, although both the PARP1 and the MRN complex appear to play important roles [56]. PARP1 has been well described as an important player of the BER/SSBR pathway responsible for the recruitment of the XRCC1-Ligase3 complex to stimulate repair [57]. PARP1 has been reported to compete for free DNA ends with Ku and to interact with ATM [58,59]. Therefore, PARP1 may contribute to the early damage response and is supposed to serve as a platform at the broken end, to recruit other factors. Our early results confirmed that the *Arabidopsis* homologs AtPARP1 and AtPARP2 are also involved in MMEJ [60]. Ku inhibits end resection, and in the absence of Ku, the MRN complex and the MRN-interacting C-terminal-binding interacting protein (CtIP) probably work together to mediate DSB resection in a-NHEJ [61]. The knockdown of Mre11 by siRNA decreased the frequency of a-NHEJ significantly without affecting the efficiency of c-NHEJ, suggesting that a-NHEJ repair of DNA DSBs requires Mre11 specifically [62]. The knockout of CtIP also results in a significant reduction in a-NHEJ [63,64]. Human Pol λ and Pol β assist MMEJ using terminal microhomology regions [65], and the interaction between PNKP and XRCC1 [66] suggests that they may engage in end processing during a-NHEJ. Furthermore, Polymerase θ (encoded by the *Polq* gene) which belongs to the DNA polymerase A family, plays an important role in the end processing process and is the key factor in TMEJ [48]. In studies on *mus308*, the *Drosophila melanogaster* homolog of *Polq*, McVey and his co-workers were the first to identify Pol θ as a factor in the a-NHEJ pathways of DSB repair [67]. Subsequently, the role of Pol θ in a-NHEJ/MMEJ has been characterized in several organisms. Biochemical studies have shown that the polymerase domain of Pol θ is able to independently carry out all of the major stages of MMEJ in vitro [68]. a-NHEJ is facilitated by sequence microhomology, which is an important signature of Pol θ-mediated end-joining. Pol θ can use a minimum of 2 bp and, optimally, 4 bp microhomology for efficient and processive DNA synthesis [69]. A second feature of TMEJ is the production of templated DNA insertions at the DSB repair junction, which can arise from sequences directly adjacent to the resected ends or other chromosomes. In addition, it has been shown that Pol θ is able to favor the a-NHEJ, by employing its ATPase activity to counteract RPA binding and promote the annealing of resected DNA substrates [70]. Recently, TMEJ has moved to the forefront of a-NHEJ, and has been raised as an indispensable player in controlling genome stability [71]. However, whether TMEJ now takes the place of MMEJ or if TMEJ is a third pathway in addition to MMEJ remains to be further determined, although several reports show that TMEJ also functions in the presence of other DSB repair pathways [72,73,74,75]. In plants, the *Arabidopsis thaliana* ortholog TEBICHI (Teb) is involved in DNA replication, recombination and gene expression [76]. A recent report demonstrated that Arabidopsis Pol θ participates in the repair of replication-associated DNA damage [77]. In mammalian cells, the XRCC1-Ligase3 complex seems to contribute to DSB ligation in the a-NHEJ pathway [78,79]. Since plants are lacking a Ligase 3 homolog, there must be other factors to take over ligation during a-NHEJ in plants. One of the candidates is SSB repair factor Ligase 1, which is associated in the a-NHEJ repair pathway in Arabidopsis [80]. Ligase 1 displays functional redundancy with Ligase 3 and might cooperate in a-NHEJ in mammals [81,82].

## 5. DSB Repair Pathway and *Agrobacterium*-Mediated T-DNA Integration

*Agrobacterium tumefaciens* is nowadays employed as a vector to create genetically modified plants. During the process of *Agrobacterium*-mediated genetic transformation, T-DNA is transferred from its tumor-inducing plasmid to the host cell’s nuclear genome. T-DNA is at random positions in the plant genome, which may lead to mutation and position effects altering the expression of the transgenes. Therefore, there is great interest in developing methods for the controlled and targeted integration of T-DNA. In yeast, this can be accomplished by providing a segment of yeast-homologous DNA in the T-DNA. The HR machinery of yeast then mediates integration at the homologous site [83]. In plants, homologous recombination can occur between a chromosomal locus and a homologous T-DNA introduced via *Agrobacterium* [84], but only with a very low efficiency. Two possible models have been recommended for T-DNA integration [85,86]. In the strand-invasion model, T-DNA integration counts on the microhomology between T-DNA and plant DNA sequences. Single-stranded T-DNA is expected to facilitate the integration [87,88,89]. In the DNA DSB repair model, the single-stranded T-DNA is first converted to a double-stranded T-DNA, whereafter this double strand form integrates into the genome at DSB sites [90]. This was supported by the fact that DSBs are preferential targets for T-DNA integration and that T-DNA can be cut by a restriction enzyme before integration [91,92]. Furthermore, by using CRISPR technology to induce DSBs, a recent finding demonstrated that T-DNA was inserted into the break sites of CRISPR/Cas9 targets with high frequency [93]. In addition, given that the microbial pathogens are capable of triggering host DNA double-strand breaks [94], the inoculation by *Agrobacterium* perhaps induces DSBs in host plant genomes as well, to favor the integration of T-DNA.

**Figure 3:**
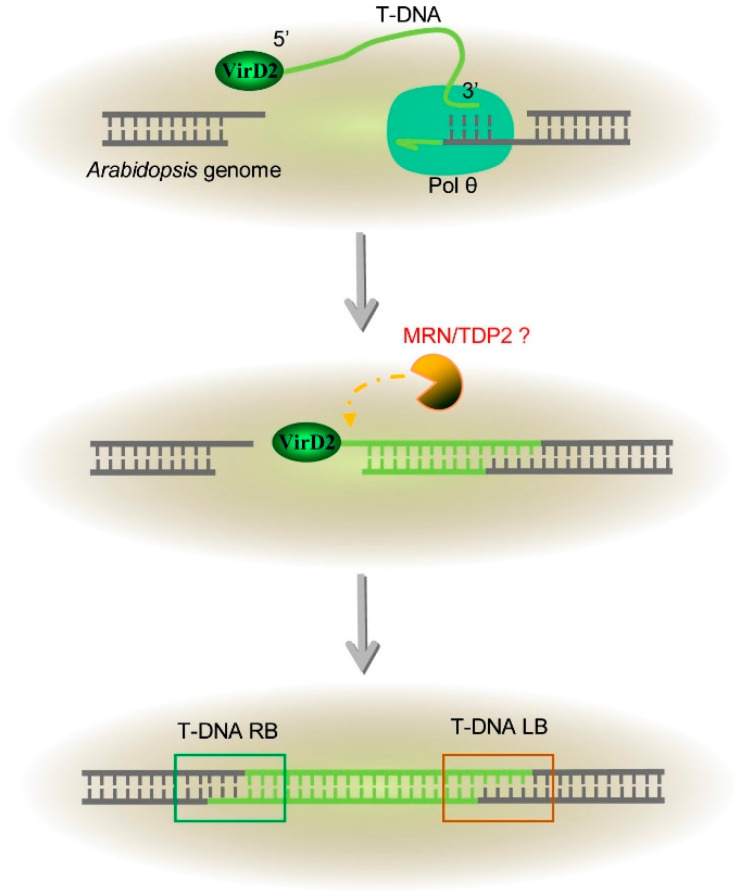
Simplified model of T-DNA integration: polymerase θ is required for capture of the T-DNA’s 3′ end; MRN or TDP2 helps to remove VirD2 for genomic capture of the 5′ end.

Although T-DNA integration has been studied for decades, its molecular mechanism has remained unclear. It should be certain that host proteins, rather than *Agrobacterium* proteins, are responsible for the T-DNA integration in plant genomes, which refer to the plant DNA-repair pathways. Therefore, the NHEJ pathways which have been described above are proposed to play an essential role in the integration of T-DNA to the plant genome. Earlier reports indicated that yeast (*S. cerevisiae*) T-DNA integration depends on NHEJ proteins, such as Ku70 and DNA ligase 4 [95,96]. NHEJ mutants in Arabidopsis were later investigated for T-DNA integration in plants. However, different research groups had conflicting results on plant T-DNA integration and revealed either no or limited negative effects [96,97,98,99]. When the multiple DNA-repair pathways were disabled at the same time, T-DNA integration was severely compromised but remained possible [100,101,102]. Nevertheless, disruption of these known repair pathways did not eliminate the end-joining, suggesting that plant T-DNA integration was mediated by other unknown proteins and pathways. Indeed, a recent study from van Kregten et al. presented that the Arabidopsis Pol θ is crucial for T-DNA integration [74]. Arabidopsis Pol θ mutants are completely recalcitrant to *Agrobacterium*-mediated transformation. As mentioned above, Pol θ is recognized as an important factor in the a-NHEJ of DSB repair due to its special properties of microhomology usage and template switching. These characteristics are also frequent at the T-DNA integration junctions [74,103]. ‘Filler DNA’ sequences, or alleged templated insertions, are often found at junctions and genomic sequences, which are often templated from the flank. Pol θ is proposed to extend paired 3′ overhangs at DNA synapses and use the opposing overhang as a template in *trans* to stabilize the DNA synapse [68]. Thus, the T-DNA left border (LB), which is a 3′ end, is the preferred substrate for Pol θ to be minimally base-paired with a 3′ end of DSB in the plant genome. Taken together, TMEJ mediates the capture of T-DNA into a DSB, providing an answer for how the T-DNA left border (LB) attaches to the plant genome (Figure 3).

Even though TMEJ explains the connecting of the T-DNA 3′ end of the integration progress, questions remain about the attachment of the T-DNA 5′ right border (RB). More recently, Nishizawa-Yokoi et al. showed that mutation of Pol θ in rice allowed stable transformation with low frequency, in which the junction fragments displayed similar characteristics to those of the wild-type plants, arguing for genes other than Pol θ in rice may also be responsible for T-DNA integration [104]. Notably, our recent results revealed that plant T-DNA integration requires Mre11 or TDP2 to remove the end protection from VirD2, to allow the capture of the T-DNA 5′ end in Arabidopsis [105]; this may fill a major gap in our understanding of T-DNA integration in plants.

## 6. DSB Repair Pathway and Gene Targeting

Gene targeting (GT) is a powerful genetic technique to change or replace endogenous genes depending on homologous recombination, which has been widely used to study gene function. Several approaches were established to select and detect GT events, including gene-specific selection (GSS) and positive–negative selection (PNS). In GSS schemes, an endogenous target gene is replaced by a copy of the same gene with a selectable mutation. In PNS schemes, the selection of homologous recombination relies on positive and negative selectable markers installed within and outside the homologous sequence, respectively. The PNS-based approach has proven to be very successful and is also used in plant species such as rice and Arabidopsis. GT can be achieved efficiently, especially in yeast and a few other organisms. However, GT is usually inefficient in the cells of multicellular eukaryotes, especially in those of plants, due to a much lower efficiency of HR than NHEJ [106]. The observed GT frequencies from multiple studies in plants are low and often in the 10^−2^ to 10^−3^ range [107,108,109,110,111]. In order to establish a feasible tool for GT, two options were tested to enhance the GT frequency based on the mechanism of HR. The first option was to promote the HR pathway by either increasing the synthesis of proteins involved in HR [112,113] or by inhibiting the synthesis of proteins involved in the NHEJ pathway [100]. The prevention of fungi NHEJ by deletion of Ku or Lig4 resulted in very efficient GT [96,114,115]. In plants, several reports show that GT efficiency can also be increased by blocking the NHEJ pathway or enhancing the HR pathway [116,117,118]. However, recent studies in mammalian and Arabidopsis indicate that the deficiency of Pol θ does not increase GT events [119,120]. Another approach was to enhance GT by inducing genomic DSBs at the target site, which became possible by the development of different classes of artificial nucleases (see below). In this way, the frequency of GT was increased significantly in different organisms, including plants [121,122,123,124]. GT was also achieved in *Arabidopsis thaliana* by expression of a site-specific endonuclease that cuts not only within the target, but also the chromosomal transgenic donor (*in planta* GT), leading to an excised targeting vector in each plant cell [125]. Due to recent developments, especially the application of a CRISPR/Cas nuclease system, several groups have achieved efficiency improvements of GT in plants. For instance, the group of Puchta used an egg-cell-specific promoter of the SaCas9 nuclease, which enabled them to sufficiently enhance GT efficiencies up to 1–6% in Arabidopsis [126]. Voytas and his co-workers employed geminivirus-based DNA replicons combined with Cas9, resulting in GT frequencies of ~1% in wheat [127]. The group of Levy also found that GT efficiency was strongly increased by using geminiviral replicons and a Cas9 system in tomato, with 25% in the T0 plant [128]. In addition, other works showed high GT rates of 9.1% in Arabidopsis and 8% in rice by using Cas9 and Cpf1, respectively, although they were based on small numbers of GT events [129,130]. Furthermore, several novel approaches seem to be applicable in plants to improve GT efficiency. The enhancing homology-directed repair (HDR) efficiency was observed by fusing Cas9 with a donor DNA sequence, which ultimately brought the donor DNA into close proximity to the DSB sites [131,132,133]. Moreover, it was interesting to find that the Cas9-CtIP fusion efficiently stimulated HDR after Cas9-mediated DNA cleavage [134]. Taken together, these findings might open a promising avenue for a higher efficiency of GT with the CRISPR/Cas9 system.

## 7. Strategies for DSB Induction

As DNA recombination events, including transgene integration and gene targeting, are increased at break sites in the genome, it has been a strategy to induce local DNA breaks to stimulate these events. Ionizing radiation (X-ray) and genotoxic chemicals (Bleomycin, MMS, etc.) were initially used to induce such DNA breaks, but as they affect the genome in an uncontrolled manner and cause mutation, this was not very successful.

Site-specific nucleases have been developed by which DSBs can be induced at a preferred site in the genome. The advent of meganucleases, such as *Sce*I, for the first time offered the possibility to induce a DSB at a specific site(s) in a large genome. Such a local break inspired a significant increase in DNA integration [90] and in gene targeting [135]. Since then, three classes of nucleases have been used extensively: zinc finger nucleases (ZFNs), transcription activator-like effector nucleases (TALENs) and the CRISPR/Cas (for ‘clustered regularly interspersed short palindromic repeats/CRISPR associated’) system.

ZFNs consist of zinc finger arrays fused to the nuclease domain of the type II restriction enzyme FokI. Each zinc finger typically recognizes three nucleotides, and engineered fingers have been combined to recognize specific longer DNA sequences. ZFNs function as a dimer to produce a DSB within the spacer between the binding sites of two ZFN monomers. ZFN-mediated gene modification has been reported in different eukaryotic organisms [121] and also in Arabidopsis [109,116,123]. Like ZFNs, TALENs are composed of DNA-binding domains and a FokI nuclease domain. Each binding domain includes a variable number of amino acid repeats, which are able to specifically recognize a single base pair of DNA [136]. TALENs and ZFNs make DSBs with 5′ overhangs. TALENs are considered to be more efficient, specific and reproducible, because TALENs are less affected by the context of targeting sequences than ZFNs, as shown in yeast [137], human [138] and Arabidopsis [139].

In 2012, an RNA-guided CRISPR/Cas nuclease system was described for inducing DNA DSBs at specific genomic loci [140]. CRISPR/Cas originates from a microbial adaptive immune system that uses RNA-guided nucleases to cleave the foreign invading sequences. The CRISPR/Cas9 used for DSB induction in eukaryote organisms is based on bacterial type II CRISPR/Cas systems, consisting of CRISPR-associated protein Cas9 and a single guide RNA chimera (sgRNA), which was engineered from the tracrRNA and crRNA [140]. Guided by the sgRNA via base-pairing to the target DNA sequence, both strands of the target DNA are cleaved by two endonuclease domains (HNH- and RuvC-like domains) of the Cas9 protein. The cleavage locations are also determined by a protospacer-adjacent motif (PAM) which is juxtaposed to the complementary region in the target DNA [140]. Furthermore, the CRISPR/Cas12a, formally known as Cpf1 belonging to the Class 2 type V CRISPR/Cas system, has emerged as an alternative and promising gene-editing tool with an efficiency that is at least comparable to the CRISPR/Cas9 [141,142]. Cas12a creates staggered DNA double-stranded break ends, while Cas9 produces blunt ends. The CRISPR/Cas system is markedly easier to design by changing the guide RNA sequence, compared to ZFNs and TALENs. It is highly specific and efficient for a vast number of cell types and organisms. Thus, CRISPR has quickly become a standard technique in genome engineering since its discovery. During the past ten years, the CRISPR technique has been applied successfully in various organisms and has shown incredibly fast development.

## 8. Concluding Remarks and Future Perspectives

Although an increasing number of proteins engaged in DNA-repair pathways have been analyzed, and their interactions investigated, in the past few decades, the repair mechanisms are not fully understood at present and require more in-depth studies. The mechanisms evolved to repair DSBs are highly conserved between organisms; however, studies in plants lag behind. These repair pathways contribute to T-DNA integration and targeted DNA insertion. The understanding of the DSB repair mechanism is beneficial to develop precise genome modification approaches, which are extremely valuable for crop plants. Furthermore, the CRISPR/Cas-mediated genome-editing tool was employed in various plants successfully in the last decade. This application not only improves our ability to deal with specific issues in fundamental research, but also adapts to creating germplasms of crop species with desired traits, and to enhancing global food security and sustainable agriculture. As we gain a deeper understanding of repair mechanisms and the improvement of nuclease-based technology, we can expect even more diversification and high-efficiency genome editing tools in the near future.

## Figures and Tables

**Table 1:** HR proteins in human, yeast and Arabidopsis.

*Homo sapiens*	*Saccharomyces* *cerevisiae*	*Arabidopsis* *thaliana*	*Arabidopsis* Gene Number	Function
Rad51	Rad51	Rad51	*At5g20850*	RecA homologue Strand invasion
MRN complex: Mre11-Rad50-Nbs1	MRX complex: Mre11-Rad50-Xrs2	MRN complex: Mre11-Rad50-Nbs1	*At5g54260* *At2g31970* *At3g02680*	DNA binding Nuclease activities DSB end processing DNA-damage checkpoints
CtIP	Sae2	Com1	*At3g52115*	DSB end processing DNA strand transition
Exo1	Exo1	Exo1A Exo1B	*At1g29630* *At1g18090*	DSB end processing
BLM	Sgs1	RecQ4A RecQ4B	*At1g10930* *At1g60930*	DSB end processing RecQ helicases
RPA1 RPA2 RPA3	RPA1 RPA2 RPA3	RPA1 RPA2 RPA3	*At2g06510* *At4g19130* *At5g45400* *At5g08020* *At5g61000*	ssDNA binding
Rad51B-Rad51C Rad51C-XRCC3 Rad51D-XRCC2	Rad55-Rad57	Rad51B-Rad51C Rad51C-XRCC3 Rad51D-XRCC2	*At2g28560* *At2g45280* *At5g57450* *At1g07745* *At5g64520*	ssDNA binding Recombination mediator
Rad52	Rad52	Rad52	*At1g71310* *At5g47870*	ssDNA binding and annealing Recombination mediator Interacts with Rad51 and RPA
BRCA1	− ^1^	BRCA1	*At4g21070*	Checkpoint mediator Recombination mediator
BRCA2	− ^1^	BRCA2-1 BRCA2-2	*At5g01630* *At4g00020*	Recombination mediator
Rad54	Rad54	Rad54	*At3g19210*	ATP-dependent dsDNA translocase Stimulates the D-loop reaction
FancM	Mph1	FancM	*At1g35530*	Helicase activity Dissociates D-loop formation and facilitates single-strand annealing

^1^ No yeast equivalent has been identified.

**Table 2:** NHEJ proteins in human, yeast and Arabidopsis.

*Homo sapiens*	*Saccharomyces* *cerevisiae*	*Arabidopsis* *thaliana*	*Arabidopsis* Gene Number	Function
Ku70/Ku80	Ku70/Ku80	Ku70/Ku80	*At1g16970* *At1g48050*	DSB end binding and protection
DNA-PKcs	− ^1^	− ^1^		protein kinase
Artemis	Snm1/PSO2	Snm1	*At3g26680*	DNA end processing
MRN complex: Mre11-Rad50-Nbs1	MRX complex: Mre11-Rad50-Xrs2	MRN complex: Mre11-Rad50-Nbs1	*At5g54260* *At2g31970* *At3g02680*	DNA binding Nuclease activities DSB ends processing DNA-damage checkpoints
PNKP	Tpp1	ZDP	*At3g14890*	DNA end processing
Pol λ	− ^1^	Pol λ	*At1g10520*	DNA polymerase DNA end processing
53BP1	Rad9	Rad9	*At3g05480*	DNA end processing
DNA ligase IV	Dnl4	lig4	*At5g57160*	ATP-dependent DNA ligase
XRCC4	Lif1	XRCC4	*At3g23100*	complex with lig4
XLF/Cernunnos	Nej1	− ^1^		complex with lig4/XRCC4
Parp1	− ^1^	Parp1	*At2g31320*	DNA end binding NAD + ADP-ribosyltransferase
Parp2	− ^1^	Parp2	*At4g02390*	DNA end binding NAD + ADP-ribosyltransferase
Parp3	− ^1^	Parp3	*At5g22470*	DNA end binding NAD + ADP-ribosyltransferase
CtIP	Sae2	Com1	*At3g52115*	DNA end processing
DNA ligase III	− ^1^	− ^1^		ATP-dependent DNA ligase
XRCC1	− ^1^	XRCC1	*At1g80420*	complex with lig3
Pol Q	− ^1^	Pol θ (Tebichi)	*At4g32700*	DNA polymerase DNA end processing

^1^ No yeast or Arabidopsis equivalent has been identified.

## Data Availability

Not applicable.
